# A soil bacterium alters sex determination and rhizoid development in gametophytes of the fern *Ceratopteris richardii*

**DOI:** 10.1093/aobpla/plz012

**Published:** 2019-03-14

**Authors:** Michael T Ganger, Rachel Hiles, Haley Hallowell, Lauren Cooper, Nicole McAllister, Doug Youngdahl, Jeremy Alfieri, Sarah J Ewing

**Affiliations:** 1 Department of Biology, Gannon University, Erie, PA, USA; 2 Department of Botany and Plant Pathology, Purdue University, West Lafayette, IN, USA; 3 Department of Biology, Auburn University, Auburn, AL, USA; 4 St. George’s University, University Centre, West Indies, Grenada; 5 Department of Microbiology and Molecular Genetics, University of Pittsburgh, Pittsburgh, PA, USA

**Keywords:** *Ceratopteris richardii*, gametophyte, plant growth-promoting rhizobacteria, *Pseudomonas nitroreducens*, rhizoid development, sex determination

## Abstract

Gametophytes of the fern *Ceratopteris richardii* develop into either hermaphrodites or males. As hermaphrodites develop, they secrete antheridiogen, or A_CE_, into the environment, inducing male development in undifferentiated gametophytes. Hermaphrodites are composed of archegonia, antheridia, rhizoids and a notch meristem, while males consist of antheridia and rhizoids. Much of the research on sexual and morphological development concerns gametophytes grown in sterile environments. Using biochemical and molecular techniques we identify a soil bacterium and explore its effects on sexual and rhizoid development. Hermaphrodite and male gametophytes were exposed to this bacterium and the effects on sexual development, rhizoid length and rhizoid number were explored. The bacterium was identified as a pseudomonad, *Pseudomonas nitroreducens*. Gametophytes grown in the presence of the pseudomonad were more likely to develop into hermaphrodites across all gametophyte densities. Across all gametophyte sizes, hermaphrodites had rhizoids that were 2.95× longer in the presence of the pseudomonad while males had rhizoids that were 2.72× longer in the presence of the pseudomonad. Both hermaphrodite and male gametophytes developed fewer rhizoids in the presence of the pseudomonad. Control hermaphrodites produced 1.23× more rhizoids across all gametophyte sizes. For male gametophytes grown in the absence of the pseudomonad, the rate of increase in the number of rhizoids was greater with increasing size in the control than the rate of increase in males grown in the presence of the pseudomonad. The pseudomonad may be acting on gametophyte sexual development via several potential mechanisms: degradation of A_CE_, changes in nutrient availability or phytohormone production. The pseudomonad may also increase rhizoid number through production of phytohormones or changes in nutrient availability.

## Teaser

The multicellular haploid generation of plants (gametophyte) is responsible for sperm and egg production. In the fern *Ceratopteris richardii*, gametophytes are free-living and may develop into either males or hermaphrodites. This developmental decision is not genetically programmed, but instead is environmentally determined. A pheromone released by hermaphrodites called antheridiogen induces individuals to develop as males. The presence of the bacterium *Pseudomonas nitroreducens* blocks male induction and results in more individuals developing as hermaphrodites. The bacterium also induces longer but fewer rhizoids to develop in both males and hermaphrodites.

## Introduction

The soil environment that natural populations of plants are exposed to is quite complex (Torsvik *et al.* 1996; Torsvik and Øvreås 2002). Bacteria represent major players in the soil with cell numbers thought to approach 1 × 10^9^–1.5 × 10^10^ per g of soil (Torsvik *et al.* 1990; Ranjard *et al.* 2000) constituting over 4000 unique genomes per g of soil (Torsvik *et al.* 1990). These bacteria are known to participate heavily in nutrient cycling, as well as intimate crosstalk with sporophyte roots (Brencic and Winans 2005). The bacteria and roots communicate through both chemical and hormonal signals (Radutoiu *et al.* 2003), which can directly initiate changes in plant behaviour and morphology (Lambrecht *et al.* 2000; Baca and Elmerich 2007; Nadeem *et al.* 2016).

The crosstalk between the sporophyte root and bacteria and between sporophyte roots themselves are well studied (Bais *et al.* 2004). Less explored are the potential relationships between free-living gametophytes and soil bacteria, although the resident bacteria for many bryophytes have been described (Opelt *et al.* 2007; Bragina *et al.* 2012). The results of such work with bryophytes, though largely correlative, strongly suggest a relationship between specific groups of bacteria and bryophytes (Opelt *et al.* 2007; Bragina *et al.* 2012). Ferns offer a unique opportunity to explore such associations given that they offer a free-living gametophyte in common with bryophytes and a free-living sporophyte generation in common with seed plants. In ferns these two generations may briefly share the same microenvironment though there are inherent differences between gametophyte rhizoids and sporophyte roots: rhizoids are multicellular, while the morphologically and functionally similar root hairs of the sporophyte are single celled (Jones and Dolan 2012).

Among some of the homosporous ferns, gametophyte sex is strongly influenced by the presence or absence of a secreted hormone in the soil called antheridiogen (Banks 1999). This system was first described by Döpp (1950, cited in Schneller *et al.* 1990) in *Pteridium aquilinum*. Since then, many others have described antheridiogen-based sex determination systems in a variety of homosporous ferns (reviewed in Yamane 1998), including one of the most studied ferns, *Ceratopteris richardii*, which serves as a model system (Hickok *et al.* 1995). The default sexual development in *C. richardii* is as a hermaphrodite; as hermaphrodites develop, they secrete antheridiogen, or A_CE_, into the environment (Banks *et al.* 1993; Eberle *et al.* 1995; Banks 1997, 1999). Developing gametophytes that perceive A_CE_ during a narrow induction window, from approximately day 3 to day 6 of development, are biased to develop as males (Banks 1999). Males require the continued presence of A_CE_ to remain male; with the removal of A_CE_, males may convert to hermaphrodites (Banks *et al.* 1993; Juarez and Banks 1998; Cheruiyot and Schwartz 2007; Ganger *et al.* 2015). Hermaphrodites are larger than males having a cordate thallus that is dorsoventrally flattened with rhizoids forming at the basal end (Nayar and Kaur 1971).

The bulk of studies on homosporous ferns, including *C. richardii*, rely on laboratory observations, while a small number have focused on the antheridiogen system in natural populations (Schneller *et al.* 1990). Where results between laboratory and natural populations have been compared, differences have been observed in the proportion of gametophytes in the population belonging to one gender class or another, with laboratory populations having higher proportions of asexuals or males (Schneller 1979; Ranker and Houston 2002). Some of these differences could be ascribed potentially to differences in the retention of antheridiogen in an agar-based environment or lack of age structure (Ranker and Houston 2002). Given the complexity of the soil environment with respect to bacterial communities and the reported associations between unique bacterial communities and bryophytes, it is possible that soil bacteria may affect the development of fern gametophytes. The objective of this study was to determine the effect of a soil bacterium on sex determination and rhizoid development in *C. richardii*.

## Materials and Methods

### Isolation and identification of soil bacteria

Soil bacteria were isolated from fern species local to Erie, PA, *Dryopteris intermedia*, *Onoclea sensibilis*, *Osmunda cinnamomea* and *Osmunda regalis*. Frozen stocks of each isolated bacterium were prepared with glycerol and maintained at −80 °C. One bacterial isolate from *O. sensibilis* that grew on *Pseudomonas* selective agar was subject to further testing. The isolate was inoculated in 5 mL of sterile tryptic soy broth (TSB) and grown overnight at 37 °C. A 2 μL aliquot of bacterial culture was added to 20 μL of Buffer BR-A (BacReady™ PCR Multiplex System, Genescript). Genomic DNA was amplified by PCR using GoTaq Green PCR Master Mix (Promega) and universal primers for the 16S rRNA gene (27FACA and 1492RT). PCR products were purified by ethanol precipitation, quantified and sequenced using 27FACA and 1492RT primers by MWG Operon. Complete 16S rRNA sequences were generated and used as input to identify the bacterial isolate using the EZTaxon-e Database (Kim *et al.* 2012) and RDP Classifier (Wang *et al.* 2007). Additional biochemical tests for pseudomonads were performed as described in Watanabe *et al.* (1977) to support identification of the isolated soil bacteria.

### Inoculation of C-Fern agar plates with bacteria

Tubes of TSB were inoculated with the identified pseudomonad from the frozen stock. Bacterial cultures were grown for 72 h at 37 °C and diluted with sterile TSB to produce cultures with an absorbance of 0.2 at 600 nm. C-Fern agar was prepared by combining C-Fern powdered media (Carolina Biological Supply Company) with granulated agar (BD Difco agar) following manufacturer’s protocols. Cooling C-Fern agar plates (55 °F, 40 mL) were inoculated with either 40 μL of the diluted pseudomonad culture (treatment) or 40 μL of sterile TSB (control). Since the bacteria are added while the agar is still liquid to produce a pour plate, bacteria end up suspended within the agar and not on top of it.

### Plating and germination of *C. richardii* spores

*Ceratopteris richardii* wild-type spores were obtained from Carolina Biological Supply Company and were diluted in 5 mL of sterile water following manufacturer’s instructions. Control and bacteria-containing plates were inoculated with *C. richardii* spores at volumes indicated in each experiment and placed under 12-h grow lights (60 μM photons m^−2^ s^−1^) supplemented with 2–15 W incandescent bulbs at 28 °C in a Percival Scientific growth chamber.

### Effects of bacteria on *C. richardii* sex determination

Eleven bacteria-containing and 11 control C-Fern agar plates were prepared as described and each was inoculated with a different volume of *C. richardii* wild-type spores: 60, 72, 85, 96, 108, 120, 132, 144, 156, 168 and 180 μL. *Ceratopteris richardii* is a model system for developmental studies, in part, due to its short life cycle (Hickok *et al.* 1987). Sexual maturity is reached in 10–12 days at temperatures used in these experiments (Spiro and Knisely 2008) and therefore, after 3 weeks, gametophytes could easily be classified as male or hermaphrodite. The proportion of hermaphrodites was determined as the number of hermaphrodites/the total number of gametophytes. This experiment was repeated a second time and analysed separately [see Supporting Information].

Using SPSS software (IBM Corp. 2017) an analysis of covariance (ANCOVA) was used to determine if the proportion of hermaphrodites differed among treatments. The total number of gametophytes per dish served as the covariate. The homogeneity of slopes assumption was tested as the interaction between the covariate and the treatment effect. Where the interaction was not significant, the analysis was run again without the interaction term following the ANCOVA protocol.

### Effects of bacteria on rhizoid growth and thallus area

Seven replicate bacteria-containing and seven replicate control C-Fern agar plates were prepared as described and inoculated with *C. richardii* wild-type spores to yield a density of ~9 gametophytes per cm^2^. Beginning 9 days after spore sowing and continuing through day 19, gametophytes were randomly chosen for image analysis using a random number table and a polar coordinate system given to each plate. Random numbers allowed for random selection of both plates and gametophytes. Day 9 was chosen because at this point it was possible to determine the gender of gametophytes, and day 19 was chosen because at this point gametophytes were often too large to be photographed using existing magnification. On each day, still images of eight males and eight hermaphrodites from at least four Petri dishes per treatment allowed for determination of gametophyte area and maximum length of rhizoids using image analysis software (Infinity Analyze, Lumenera Corporation). Because hermaphrodites are dorsoventrally flattened and males are only a couple of cell layers thick, size of gametophytes can be determined by measuring gametophyte area. The number of rhizoids was determined by counting. Since gametophytes are known to communicate with and potentially influence one another, the sizes of gametophytes are not independent. Therefore, in order to avoid pseudoreplication (Hurlbert 1984), measurements of hermaphrodites and males were averaged separately for each Petri dish [see Supporting Information].

Using SPSS software (IBM Corp. 2017), a two-factor multiple analysis of covariance (MANCOVA) was used to determine factors affecting the growth of rhizoids. Number and maximum length of rhizoids were the dependent variables, while gametophyte gender and bacterial treatment served as main effects. Both maximum rhizoid length and rhizoid number are expected to increase with gametophyte area and therefore gametophyte area was used as a covariate. The two dependent variables and the covariate were log-transformed to improve normality. The three-way interaction between the covariate, gender and bacterial treatment was included in the model along with the tests of homogeneity of slopes: interactions between each factor and the covariate. With non-significant homogeneity of slopes tests, the model was rerun without these terms. With significant interactions, the model was broken into separate analyses.

In order to determine if hermaphrodite thallus area differed for *C. richardii* grown in the presence and absence of the pseudomonad, average thallus area was determined for each treatment on each day from day 9 to day 17 [see Supporting Information]. Using SPSS software (IBM Corp. 2017), an ANCOVA was performed on hermaphrodite thallus area for *C. richardii* grown in the presence and absence of the pseudomonad. Day served as a covariate and the interaction between day and log(area of hermaphrodite) served as a test of the homogeneity of slopes assumption.

### Effects of bacteria on *C. richardii* germination rates

Ten replicate bacteria-containing and 10 replicate control C-Fern agar plates were prepared as described and inoculated with *C. richardii* wild-type spores to yield a density of ~9 gametophytes per cm^2^. Cumulative germination rates were determined by following an average of 28 spores for 8 days. This was made possible by frame-grabbing an image of a section of the plate. Subsequent viewings of the plate were aligned with the saved image ensuring that the same spores were followed over time. Germination rates were determined for each treatment on each day from day 3 to day 8 [see Supporting Information]. Using SPSS software (IBM Corp. 2017), a repeated measures analysis of variance (ANOVA) was performed to compare germination rates for *C. richardii* grown in the presence and absence of the pseudomonad.

## Results

### Soil bacteria

A soil isolate collected from the fern *O. sensibilis* was observed to grow on *Pseudomonas* selective agar. Comparison of the 16S rRNA sequence from the soil bacteria isolate to the EZTaxon-e Database resulted in a sequence alignment of 29 out of 30 results to bacteria within the genus *Pseudomonas*, where the top sequence alignment was to *Pseudomonas nitroreducens* with a similarity score of 99.86 %. The results of the additional biochemical testing of the bacterial soil isolate matched the results following Watanabe *et al.* (1977; Table 1). Both 16S rRNA sequence analysis and biochemical tests support the identification of the unknown bacterium as most closely resembling *P. nitroreducens*.

**Table 1.** Results of biochemical testing of the soil bacterium. Results from Watanabe *et al.* (1977) are shown for comparison.

Test	Soil pseudomonad	Watanabe *et al.* (1977)
Colony morphology	Raised, dull, yellow	Raised, dull, yellow orange
Nitrate reduction	+	+
Nitrate respiration	+	+
H_2_S production	−	−
Mannitol, sorbitol, glucose, maltose, lactose	No gas	No gas
Indole production	−	−
Starch hydrolysis	−	−
Growth at 37 °C	+	+
Urease	−	Weakly +
Catalase	+	+

### Effects of bacteria on *C. richardii* sex determination

**Figure 1.**
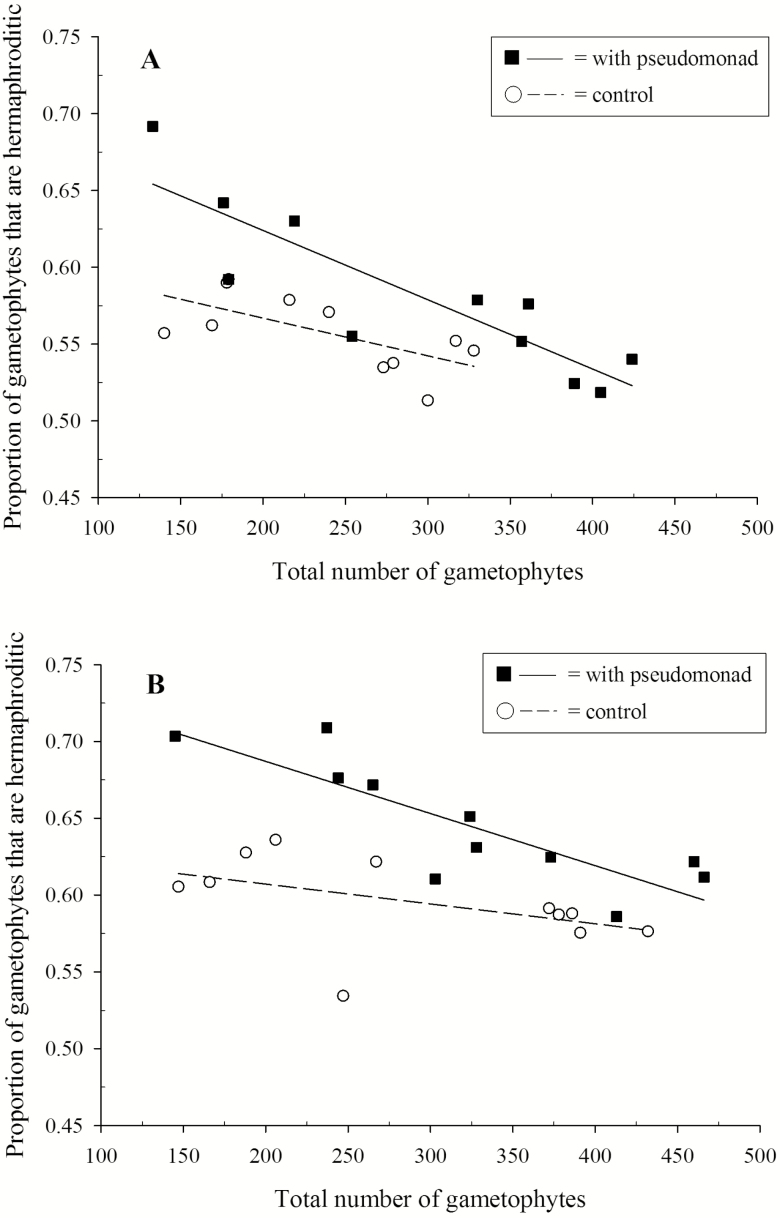
Relationship between the total number of gametophytes and the proportion of hermaphroditic gametophytes for *Ceratopteris richardii* grown in the presence and absence of the pseudomonad as determined from two separate experiments.

In order to determine whether the pseudomonad affected sexual development in *C. richardii*, an ANCOVA was performed. In order to determine whether the relationship between the number of gametophytes and the percentage of hermaphrodites differed between gametophytes in the presence and absence of the pseudomonad, the interaction between bacterial treatment and number of gametophytes was included and served as a test of the homogeneity of slopes assumption. The interaction was not significant (*P* = 0.146) and therefore the model was run again without the interaction term following ANCOVA protocols. There was a significant effect of bacteria on the percentage of hermaphrodites (*F*_1, 19_ = 17.30, *P* = 0.001; Fig. 1A). Across densities, the presence of the bacteria resulted in a higher proportion of hermaphrodites. The percentage of hermaphrodites decreased with an increase in total gametophyte number (*F*_1, 19_ = 38.60, *P* < 0.001; Fig. 1A). For the repeat experiment the interaction between bacterial treatment and number of gametophytes served as a test of the homogeneity of slopes assumption. The interaction was not significant (*P* = 0.07) and therefore the model was run again without the interaction term. There was a significant effect of bacteria on the percentage of hermaphrodites (*F*_1, 19_ = 24.771, *P* < 0.001; Fig. 1B). Across densities, the presence of the bacteria resulted in a higher proportion of hermaphrodites. The percentage of hermaphrodites decreased with an increase in total gametophyte number (*F*_1, 19_ = 15.415, *P* < 0.005; Fig. 1B).

### Effects of bacteria on rhizoid growth

**Figure 2.**
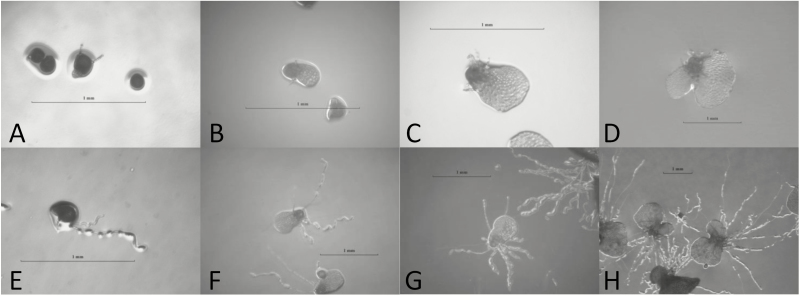
Images of control *Ceratopteris richardii* gametophytes 10 (A), 13 (B), 15 (C) and 20 (D) days from sowing, and gametophytes grown with the pseudomonad 10 (E), 13 (F), 15 (G) and 20 (H) days from sowing. Scale bars on each image are 1 mm.

Both hermaphroditic and male gametophytes showed dramatic changes in rhizoid development over all days of the experiment (Fig. 2). In order to determine the effect of the pseudomonad on rhizoid development, a MANCOVA was performed with Pillai's trace used as the test statistic. Pillai's trace values range from 0 to 1 with increasing values indicating that the effects are contributing more to the overall statistical model. An interaction between gametophyte and log(gametophyte area) (Pillai's trace = 0.277, *F*_2, 127_ = 24.32, *P* < 0.001) served as the test of the homogeneity of slopes assumption and its significance precluded an overall analysis using both hermaphrodites and males together. Therefore, two separate MANCOVAs were performed, one for males and one for hermaphrodites.

**Figure 3.**
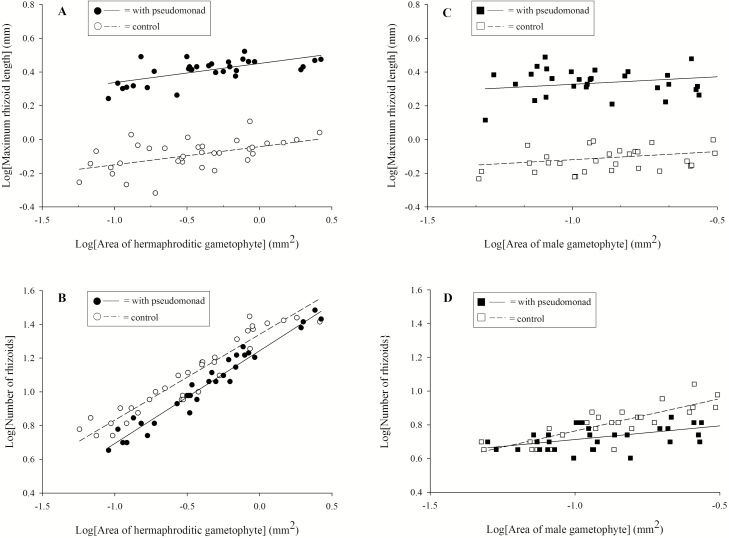
Plots of rhizoid growth and gametophyte area: (A) relationship between the log[area] and log[maximum rhizoid length] for *Ceratopteris richardii* hermaphrodites grown in the presence and absence of the pseudomonad; (B) relationship between the log[area] and log[number of rhizoids] for *C. richardii* hermaphrodites grown in the presence and absence of the pseudomonad; (C) relationship between the log[area] and log[maximum rhizoid length] for *C. richardii* males grown in the presence and absence of the pseudomonad; and (D) relationship between the log[area] and log[number of rhizoids] for *C. richardii* males grown in the presence and absence of the pseudomonad.

For the hermaphrodite MANCOVA, the interaction between bacterial treatment and log(gametophyte area) served as the homogeneity of slopes test of whether the relationship between log(gametophyte area) and rhizoid development differed between hermaphrodites in the presence and absence of the pseudomonad. No interaction was evident (Pillai's trace = 0.02, *F*_2, 65_ = 0.655, *P* = 0.523) and therefore, the model was rerun without this interaction term. Bacterial treatment predicted rhizoid attributes (Pillai's trace = 0.936, *F*_2, 66_ = 480.71, *P* < 0.001, canonical coefficient = 0.967). The new variate, a canonical variate that is a linear combination of the two rhizoid measures, was composed mainly of log(maximum rhizoid length) (0.970) and less of log(rhizoid number) (−0.205). Canonical coefficients, measurements of the strength of the relationship between canonical variates, had opposite signs indicating that maximum rhizoid length was positively related to the presence of the bacterium, while the number of rhizoids was negatively related. The new variate correlated most strongly with log(maximum rhizoid length) (0.979) and less so with log(rhizoid number) (−0.249), and therefore, univariate plots are presented (Fig. 3A and B). The longest rhizoid developed by hermaphrodites was ~295 % longer in the presence of the bacteria, while hermaphrodites produced 123 % more rhizoids in the control.

The log(gametophyte area) predicted rhizoid attributes (Pillai's trace = 0.932, *F*_2, 66_ = 455.24, *P* < 0.001). The new variate, a linear combination of the two rhizoid measures, was strongly composed of the number of rhizoids (0.984) and less so the maximum length of rhizoids (0.228). The new variate correlated strongly with the number of rhizoids (0.974) rather than with the maximum length of rhizoids (0.184). Canonical coefficients were of similar sign, indicating that both log(rhizoid number) and log(maximum rhizoid length) increase with increasing gametophyte area.

For the male MANCOVA, the homogeneity of slopes assumption was tested using the interaction between bacterial treatment and log(gametophyte area) and determined whether the relationship between log(gametophyte area) and rhizoid development differed between males grown in the presence and absence of the pseudomonad. This interaction was significant (Pillai's trace = 0.157, *F*_2, 61_ = 5.67, *P* = 0.006) and therefore, the MANCOVA was broken into two ANCOVAs, one for log(maximum rhizoid length) and the other for log(rhizoid number). Given the overall correlation between maximum rhizoid length and rhizoid number, a critical *P*-value of 0.025 was used rather than *P* = 0.05. For log(maximum rhizoid length), the interaction between bacterial treatment and log(gametophyte area) served as a test of the homogeneity of slopes assumption to determine whether the relationship between log(gametophyte area) and log(maximum rhizoid length) differed between males grown in the presence and absence of the pseudomonad. The interaction was not significant (*F*_1, 62_ = 0.002, *P* = 0.962) and therefore, the analysis was rerun without this term. The maximum length of rhizoids produced by males in the bacterial treatment was 272 % longer than control rhizoids (*F*_1, 63_ = 492.23, *P* < 0.001; Fig. 3C). The log(gametophyte area) did not predict log(maximum rhizoid length) (*F*_1, 63_ = 3.80, *P* = 0.056). For log(rhizoid number), the interaction between bacterial treatment and log(gametophyte area) served as a test of the homogeneity of slopes assumption to determine whether the relationship between log(gametophyte area) and log(rhizoid number) differed between males grown in the presence and absence of the pseudomonad. The interaction was significant (*F*_1, 62_ = 10.86, *P* = 0.002; Fig. 3D) and therefore, the ANCOVA could not be run.

### Effect of bacteria on *C. richardii* germination rates and thalli growth

**Figure 4.**
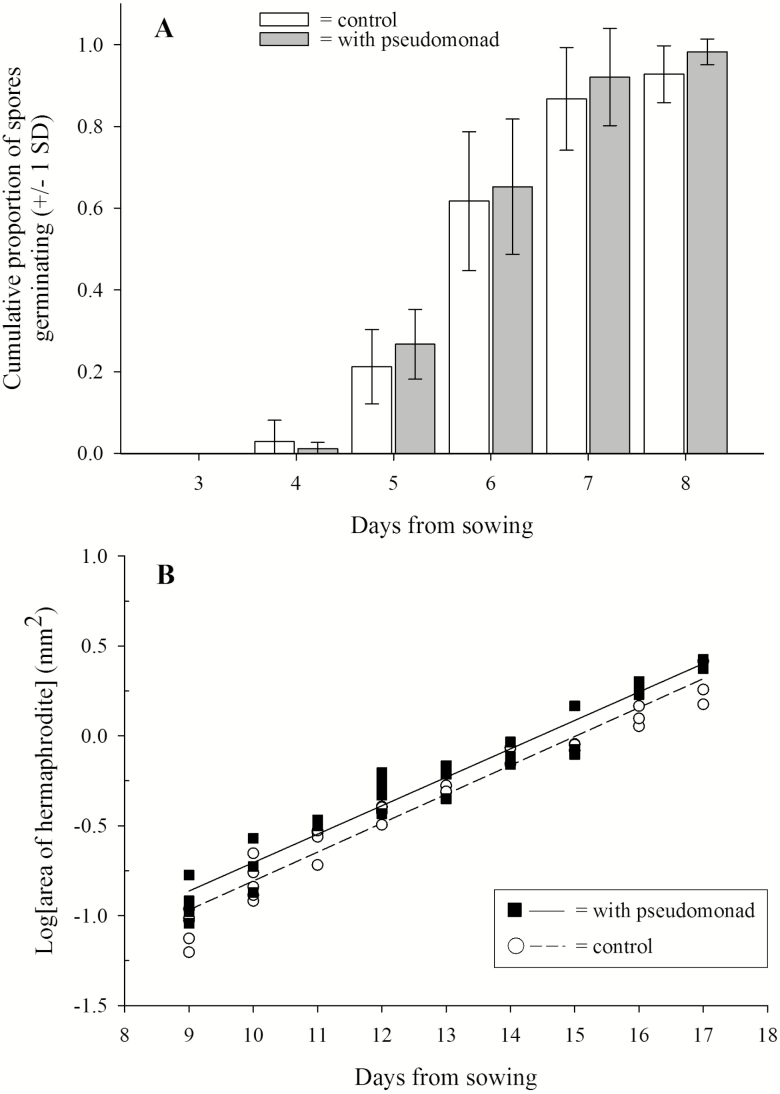
Histograms of (A) cumulative germination of *Ceratopteris richardii* spores grown in the presence and absence of the pseudomonad between 3 and 8 days post sowing and (B) relationship between the log[area] for *C. richardii* hermaphrodites and time (days 9 through 17) for gametophytes grown in the presence and absence of the pseudomonad.

In order to determine whether the pseudomonad affected germination rates, a repeated measures ANOVA was performed. Cumulative germination rates increased over the 8 days (Pillai’s trace = 0.998, *F*_5, 14_ = 1252.66, *P* < 0.001) with over half of the spores in both the treatment and control germinating, on average, within 6 days. Germination rates did not differ between *C. richardii* grown in the presence and absence of the pseudomonad (Pillai’s trace = 0.275, *F*_5, 14_ = 1.064, *P* = 0.421; Fig. 4A).

In order to determine whether thallus area differed between hermaphrodites grown in the presence or absence of the pseudomonad, an ANCOVA was performed. The interaction between day and bacterial treatment, which served as a test of the homogeneity of slopes, was not significant (*F*_1, 64_ = 0.068, *P* = 0.795) and therefore the analysis was rerun without the interaction term. Hermaphrodite thallus area was 134 % larger in *C. richardii* grown in the presence of the pseudomonad (*F*_1, 65_ = 12.149, *P* < 0.005; Fig. 4B). Hermaphrodite gametophytes increased in size over the course of the experiment (*F*_1, 65_ = 973.835, *P* < 0.001).

## Discussion

The soil environment in which plants interact is complex (Torsvik *et al.* 1996; Torsvik and Øvreås 2002). Bacterial diversity is likely to be high (Torsvik *et al.* 1990) and bacteria are known to participate in communication with plant roots (Bais *et al.* 2004). Less studied are potential interactions between soil bacteria and free-living gametophytes, such as those found in the fern lineage.

The soil bacterium used in our research is a pseudomonad best identified as *P. nitroreducens* using both 16S rRNA analysis and biochemical testing. The addition of this bacterium to cultures of *C. richardii* had profound effects on gametophyte development. The pseudomonad reduced the percentage of male gametophytes across a range of *C. richardii* densities and increased rhizoid and thalli growth, while decreasing rhizoid number in both hermaphrodite and male gametophytes.

### Sex determination

In *C. richardii*, the presence of hermaphrodite-produced antheridiogen (A_CE_) can induce male development through a process known as induction. The pseudomonad used in these experiments reduced induction rates in *C. richardii*, resulting in a higher proportion of hermaphrodite gametophytes even when density was taken into account. The effects of A_CE_ and other antheridiogens produced by other species are dosage dependent (Stevens and Werth 1999; Quintanilla *et al.* 2007; Ganger and Sturey 2012). Thus, a reduction in the concentration of A_CE_ by the pseudomonad could explain the reduced percentages of hermaphrodites observed, for example, if the bacteria were using A_CE_ as a carbon source. Other strains of *P. nitroreducens* have been shown to utilize complex molecules, such as estrogen, as a carbon source (Huang *et al.* 2014). A_CE_ is thought to be a gibberellin (Warne and Hickok 1989; Yamane 1998), and there is a great deal of similarity between the basic structure of gibberellins and estrogen, though gibberellins are inherently more similar to androgens (Chailakhyan and Khrianin 1987). Additionally, members of the genus *Pseudomonas* have been shown to degrade complex hydrocarbons (Nie *et al.* 2010; Palleroni *et al.* 2010).

Alternatively, the pseudomonad may affect sex determination by altering nutrient availability and quality. Some have argued a link between environmental quality and the likelihood of undifferentiated gametophytes developing as males. Where environmental quality, specifically nutrient availability, is low, more males would be expected to develop given the increased resource demands of being a hermaphrodite (Haig and Westoby 1988). Versions of this hypothesis have been previously tested for *C. richardii* by Ayrapetov and Ganger (2009) and Goodnoe *et al.* (2016). In both cases, no effect of nutrient concentration on induction rates was found for the concentrations and stoichiometry of nutrients that were used; however, there is some empirical support of a nutrient effect in another fern, *Woodwardia radicans* (DeSoto *et al.* 2008).

If the pseudomonad participates in nutrient cycling and frees up nutrients that would normally be unavailable to *C. richardii*, then these additional resources could affect induction rates. We do not consider this likely given that C-Fern media is widely used to culture *C. richardii* gametophytes and likely represents a high-quality resource environment. Alternatively, the bacterium may be yielding novel nutrients as the bacteria conduct metabolism or as bacteria decompose.

Gametophytes experiencing higher resource environments would likely be affected in other ways besides decreased induction rates. Growth rates of the gametophyte thallus and germination rates would be expected to increase as well. Hermaphrodite thalli were significantly larger in the presence of the pseudomonad than similarly aged thalli in the control. However, cumulative germination rates did not differ between *C. richardii* spores grown in the presence or absence of the pseudomonad for days 4–8 post sowing.

It is also possible that the bacterium identified as *P. nitroreducens* directly affects induction by releasing a molecule or molecules that function to communicate with *C. richardii* gametophytes. The pseudomonad could be producing abscisic acid (ABA). Soil bacteria, including members of the genus *Pseudomonas* (Naz and Bano 2010), have been shown to produce ABA (Karadeniz *et al.* 2006). Abscisic acid in culture increases the percentage of hermaphrodites (Warne and Hickok 1991) by acting in opposition to A_CE_, which is presumed to be a gibberellin (Warne and Hickok 1989; Yamane 1998).

### Rhizoid development

The length and number of rhizoids increased between 9 and 19 days post sowing for both hermaphrodite and male gametophytes. However, the rates of increase and the effects of the pseudomonad and gametophyte area on hermaphrodite and male development were different, and thus the two types of gametophytes are considered separately.

In hermaphrodite gametophytes the pseudomonad increased rhizoid lengths when compared to control hermaphrodites. The effect was such that hermaphrodite rhizoids in the presence of the pseudomonad were 2.95× longer on average across all thalli sizes than control rhizoids. New rhizoids were produced at a slower rate in hermaphrodites grown with the pseudomonad such that control hermaphrodites had 1.23× more rhizoids across all thalli areas. The pseudomonad appears to have caused a change in resource allocation within hermaphrodites from a larger number of shorter rhizoids to a smaller number of longer rhizoids.

In male gametophytes the effect of the pseudomonad is more complex. As with hermaphroditic gametophytes the pseudomonad increased rhizoid length in male gametophytes by 2.72× when compared to the control, a number that is similar to the increase for hermaphrodite gametophytes. The addition of new rhizoids in male gametophytes grown in the presence of the pseudomonad was slower than for control male gametophytes. This overall effect of new rhizoid suppression was not consistent across all male gametophyte sizes, but was rather stronger in larger male gametophytes. Perhaps flexibility in new rhizoid development is not possible in males to the same extent as in hermaphrodites. This may be in part due to the smaller overall resource budget of males given their much smaller overall size.

Roots have been shown to engage in optimal foraging and are known to elongate in nutrient poor habitats and proliferate in nutrient rich habitats (Hodge 2004; Kembel and Cahill 2005; de Kroon and Mommer 2006). If rhizoids, the analogous structures in gametophytes (Jones and Dolan 2012), were to follow the same model, then the lengthening observed in *C. richardii* rhizoids might be due to lower overall nutrient quality. It is possible that the pseudomonad, through nitrate reduction, is lowering the levels of nitrate available to the gametophytes. However, if lower levels of nutrients are affecting rhizoid growth, then we might expect germination rates and thalli sizes to be affected as well. As discussed above, no differences in the germination rates of spores in the presence or absence of the bacteria were found. Additionally, though thalli sizes on all days were larger in the presence of the bacteria, this is counter to what would be predicted if the pseudomonad were lowering nitrogen availability.

In fact, the *Pseudomonas* genus is one of a number of genera described as plant growth promoting (PGP; Lugtenberg and Kamilova 2009; Mitter *et al.* 2013). Members of the genus have been shown to increase root hair length in *Arabidopsis thaliana* (Contesto *et al.* 2008), increase overall biomass in *Maize* (Shaharoona *et al.* 2006) and *Oryza sativa* (Mirza *et al.* 2006), and increase root and shoot biomass in mung bean (Sharma and Johri 2003). The mechanisms behind such effects on sporophyte growth and development are not fully understood (Dey *et al.* 2004). However, one well-studied mechanism involves the bacterial production of phytohormones (Danger and Basu 1987; Patten and Glick 2002; Dobbelaere *et al.* 2003; Dey *et al.* 2004).

Many species of bacteria are known to communicate with plant roots hormonally. A large number of soil bacteria produce auxin (Patten and Glick 1996), including a strain of *P. nitroreducens* (Halda-Alija 2003) and many other species of the *Pseudomonas* genus (Glickmann *et al.* 1998; Patten and Glick 2002; Ahmad *et al.* 2005; Picard and Bosco 2005). The addition of auxin to sporophytes is known to stimulate root production, both adventitious and non-adventitious, and has been shown to increase the number of marginal rhizoids in *C. richardii* gametophytes at 2,4-dichlorophenoxyacetic acid (2,4-D) and α-napthaleneacetic acid (NAA) concentrations above 1 × 10^−6^ (Hickok and Kiriluk 1984). Auxin has also been shown to increase the length of rhizoids in *Physcomitrella* (Sakakibara *et al.* 2003), a non-vascular plant. Synthetic and natural auxins have been shown to increase both cell size and division in gametophytes (Miller 1968). Interestingly, auxin has also been shown to affect sexual development in *C. richardii* by working to reduce the percentage of hermaphrodites in culture (Hickok and Kiriluk 1984). This is in contrast to the increased percentage of hermaphrodites in the presence of the pseudomonad observed in our experiments. The increased percentage of hermaphrodites in the presence of the pseudomonad does not negate the potential for auxin to influence rhizoid development in *C. richardii*, since different concentrations and forms of auxin may have contradictory results, or perhaps auxin release and either nutrient effects or A_CE_ degradation are acting in concert. If separate mechanisms are at work on rhizoid development and sexual development, then it is likely that other soil bacteria may produce molecules that employ one but not the other mechanism, in which case it may be possible to decouple the effects on rhizoid development changes from effects on sex determination.

In addition to auxin, soil bacteria are known to produce cytokinins (Dey *et al.* 2004), ABA (Danger and Basu 1987; Dobbelaere *et al.* 2003), gibberellic acid (GA; Sivasakthi *et al.* 2013) and jasmonic acid (JA; Forchetti *et al.* 2007) and reduce ethylene production in plant roots (Li *et al.* 2000; Penrose and Glick 2001). Cytokinins have been shown to induce the formation of rhizoid initials in *C. richardii* gametophytes grown in the dark (Spiro *et al.* 2004). This effect is opposite to that seen here by gametophytes in the presence of the pseudomonad. Abscisic acid has been shown to increase rhizoid number in *C. richardii* at molarities between 1 × 10^−7^ and 1 × 10^−5^ M and inhibit their development at higher concentrations (Hickok 1983). Gibberellic acid has a relatively minor effect on gametophytic growth, while it has a major effect on sex determination (Miller 1968). Promotion of rhizoid elongation and cell division has been reported in the presence of GA, but only for low light environments (Miller 1968). Jasmonic acid has been studied in the fern *Platycerium bifurcatum* and promoted early gametophyte development by causing increased length and number of rhizoids and increased number of cells per gametophyte (Camloh *et al.* 1996).

Working with gametophytes directly in the environment is challenging due to difficulty in finding and identifying gametophytes to species (Schneider and Schuettpelz 2006). In the few studies that have explored sex determination of fern gametophytes in nature, higher percentages of hermaphrodites tend to occur (Schneller 1979; Ranker and Houston 2002). Working with fern gametophytes is made much easier by utilizing sterile culture in the laboratory. Doing so has facilitated the understanding of sex determination and rhizoid development. However, the interactions between gametophytes and bacteria in the soil paint a much more complex image of gametophyte sexual development and rhizoid development.

## Sources of Funding

This work was supported by a Cooney-Jackman Endowed Professorship to M.T.G. and the Biology Department at Gannon University.

## Contributions by the Authors

M.T.G. planned and designed the bacteria-fern experiments, conducted statistical analyses and wrote the manuscript; S.J.E. and N.M. performed the molecular identification of the pseudomonad; L.C. performed the biochemical testing of the pseudomonad; M.T.G., R.H., H.H., D.Y. and J.A. conducted the bacteria-fern experiments. All authors commented on and approved the manuscript.

## Conflict of Interest

None declared.

## Supplementary Material

Supplementary Material
